# Perceiving others’ cognitive effort through movement: Path length, speed, and time

**DOI:** 10.1177/17470218231183963

**Published:** 2023-07-03

**Authors:** Marcell Székely, John Michael

**Affiliations:** Department of Cognitive Science, Central European University, Budapest, Hungary; John Michael is now affiliated to Department of Philosophy, Universita` degli Studi di Milano Statale, Milan, Italy

**Keywords:** Social cognition, effort perception, effort, joint action, naive utility calculus

## Abstract

Effort perception is a crucial capacity underpinning characteristically human forms of sociality, allowing us to learn about others’ mental states and about the value of opportunities afforded by our environment, and supporting our ability to cooperate efficiently and fairly. Despite the crucial importance and prevalence of effort perception, little is known about the mechanisms underpinning it. Across two online experiments (*N* = 462), we tested whether adults estimate others’ cognitive effort costs by tracking perceptible properties of movement such as path length, time, and speed. The results showed that only time had a consistent effect on effort perception, that is, participants rated longer time as more effortful. Taken together, our results suggest that within the context of our task—observing an agent deciphering a captcha—people rely on the time of others’ actions to estimate their cognitive effort costs.

Effort perception—that is, the capacity to estimate the effort costs that observed agents are investing in specific ongoing activities—is a crucial capacity underpinning characteristically human forms of sociality. Effort perception enables one to estimate to what extent other agents prioritise the goals they are currently pursuing, and accordingly to anticipate their future decisions and actions. In addition, for highly cooperative species such as humans, effort perception is particularly important insofar as it provides a key input for inferences about fairness, for example, enabling us to calibrate our own effort contribution to match the effort contributions of a partner. Indeed, effort perception may prompt one to decrease one’s own effort investment to avoid being exploited, or to increase one’s effort investment to ensure an equal or fair distribution of effort costs. Moreover, accurate effort perception may also play an important supporting role in social learning: By estimating to what extent others prioritise particular goals, we can draw inferences about the value that those goals may have for us, irrespective of whether we pursue them jointly or individually.

In view of the functional advantages to be gained from accurately assessing the amount of effort that others are investing in specific activities, it is no surprise that humans continuously track others’ effort investment (Apps et al., 2016), and do so quite accurately (Liang et al., 2019), especially when the stakes are high (Ibbotson et al., 2019). Indeed, research by Gergely and Csibra (2003) shows that even infants as young as 12 months old rely on information about agents’ effort costs to infer those agents’ goals and to predict their actions. More recently, researchers have found that infants (Liu et al., 2017) and 5- to 6-year-old children (Jara-Ettinger et al., 2015) take agents’ effort costs into account to infer their preferences. Tying this research together, Jara-Ettinger et al. (2016) spell out a systematic theory of the computational principles—that is, the “naive utility calculus”—governing the attribution of goals, preferences, and other mental states as well as abilities to observed agents, based on the costs and benefits of their actions. Likewise, some recent research has documented the effects of effort perception upon adults’ and even infants’ willingness to invest effort. For example, Székely and Michael (2018) found that adult participants persisted longer on an effortful task when they had perceived a partner investing a high level of effort than when they had perceived the partner investing a low level of effort (see also Chennells & Michael, 2018). Extending these results, Székely and Michael (2023) found that adults chose to invest more or less effort to reduce inequity with respect to joint action partners’ effort investment. In the developmental literature, Leonard et al. (2017) reported that infants who observed a demonstration of an adult working hard to achieve her goal persisted longer on a novel task than infants who observed the adult succeed effortlessly.

Despite the crucial importance and prevalence of effort perception, little is known about the mechanisms underpinning it. One may speculate that we assess others’ effort costs by simple heuristics based on perceptible properties of their actions. Specifically, greater magnitude in dimensions such as path length, time, or speed may indicate greater effort costs. The rationale for this is that greater magnitudes along these dimensions typically co-vary with greater outlays of energy and may therefore be expected to be correlated with higher effort investment. Thus, by tracking such perceptible properties of actions, perceivers may be able to access information about the current effort investments of observed agents. And indeed, this assumption has been fruitfully adopted in some important research in developmental psychology (Csibra, 2008; Csibra et al., 2003, 1999; Gergely et al., 1995; Kamewari et al., 2005; Southgate et al., 2008; Verschoor & Biro, 2012; Woodward, 1998). However, it must be acknowledged that it has yet to be directly tested and has not been investigated in relation to cognitive effort perception.

In the current study, we tested whether adults estimate others’ cognitive effort costs by tracking perceptible properties of actions. In particular, we hypothesised that people expect path length, time, and speed to be positively correlated with effort costs because greater magnitude in dimensions such as path length, time, and speed typically correspond to greater outlays of energy. To test this, we implemented an effort perception task in two experiments. It is important to note that path length, time, and speed are necessarily confounded: It is impossible to simultaneously manipulate path length, time, and speed independently because speed is a linear combination of path length and time. Therefore, in the first experiment, we manipulated path length separately and speed/time together, whereas in the second experiment, we manipulated time separately and speed/path length together. This strategy enabled us to tease apart the relative contributions of each of these factors to effort perception.

## Experiment 1

**Figure 1.**
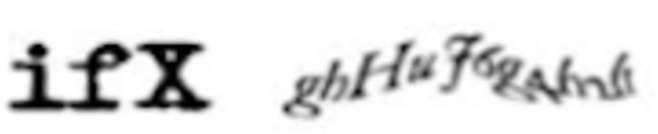
Participants were presented with examples of captchas at the beginning of the experiment. However, during the trials, they did not see the captcha or the blurry captcha itself; they only saw asterisks indicating the process of the partner deciphering each character of the captcha.

**Figure 2.**
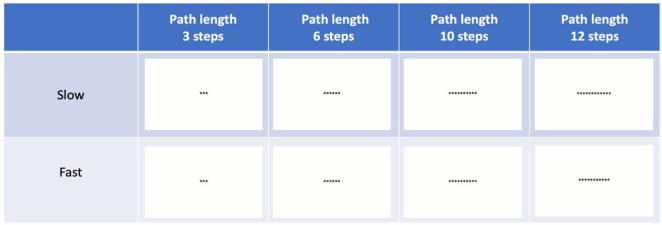
During the video, strings of asterisks appeared on the screen to indicate that an agent was solving a captcha.

To test whether people estimate others’ effort costs by tracking the speed or path length of an action, we implemented an effort perception task. In this task, participants were told that they would view recordings of a partner solving text-based captchas. A captcha is a type of cognitive task that is intended to distinguish human from machine input. In a text-based captcha, people are required to decipher a string of blurry letters. They are frequently encountered on online platforms, so we can assume that most participants are familiar with them, although they may not be familiar with the label “captcha” (see Figure 1 for two examples of text-based captchas). On each trial, a video was presented to participants in which stars progressively appeared to indicate that the partner was solving a captcha, and then they were asked how much effort they thought it had taken the partner to solve this captcha. It is important to note that it was not possible for participants to simply judge the difficulty of each deciphering action for themselves because we did not show participants the captchas that the partners were ostensibly solving. Instead, we showed them examples of captchas at the beginning of the experiment (see Figure 1); and then, on each trial, asterisks appearing on the screen indicated that the partner was entering letters/digits of the captcha (see Figure 2). In other words, participants only ever saw asterisks indicating the process of the partner deciphering each character of the captcha, but never the actual characters of the captcha, nor indeed the blurry captcha itself. Participants estimated others’ effort costs of deciphering a captcha on a Likert-type scale (1–7).

In a within-subject design experiment, we manipulated the process of deciphering the captcha by two factors: Length and Speed/Time. We manipulated Length by modifying the number of steps (characters) it takes to solve the captcha. There were four levels of captcha path length: 3, 6, 10, and 12 steps. In addition, we manipulated the Speed/Time at which these steps were taken. Captchas with equal length were completed faster, in shorter time in the Fast condition than in the Slow condition.

This design enabled us to investigate whether participants estimate others’ effort costs by tracking the path length and speed/time of an action. We predicted a main effect of Length—that is, we expected participants to estimate others’ effort costs in deciphering a captcha as higher when there were more steps. Moreover, we predicted a main effect of Speed/Time. Specifically, we predicted that if participants track speed then they should estimate others’ effort costs in deciphering a captcha as higher when it was completed more quickly; or alternatively, if participants track time, then they should estimate others’ effort costs in deciphering a captcha as higher when it was completed slower.

### Method

#### Participants

We expected a medium effect size based on pilot results, and therefore our target sample was 200 participants. Due to a technical error, we collected data from 298 participants. Of these, 39 individuals were excluded from analyses because they did not complete the task or failed two of three comprehension check questions, leaving a sample of 259 (i.e., 259 participants: 119 female, 3 other, 1 prefer not to say, *M*_age_ = 28.88 years, *SD*_age_ = 9.3 years) participants in the final data set. All participants were recruited through the Prolific recruitment platform (www.prolific.co) and were naive to the purpose of the study. All participants gave their informed consent at the start of the experiment, could withdraw from the experiment at any time, and received a fee of 90 pence for their participation. The experiment was conducted in accordance with the Declaration of Helsinki and was approved by the United Ethical Review Board for Research in Psychology (EPKEB).

#### Apparatus and stimuli

The algorithm for executing the process of solving the captcha was programmed in Python (Peirce, 2007), and it behaved in a human-like manner: Sometimes it speeded up or slowed down. The outputs of the algorithm were video recorded and embedded in a survey hosted on surveymonkey.com. Participants were required to use a desktop computer to access the task.

There were eight videos in which stars progressively appeared to indicate that an agent was solving a captcha. The first-level captchas consisted of three characters and were deciphered in 4 s in the Fast condition and in 8 s in the Slow condition. The second-level captchas consisted of six characters and were deciphered in 7 s in the Fast condition and in 14 s in the Slow condition. The third-level captchas consisted of 10 characters and were deciphered in 8 s in the Fast condition and in 16 s in the Slow condition. The fourth-level captchas consisted of 12 characters and were deciphered in 9 s in the Fast condition and in 18 s in the Slow condition. During the trials, participants only saw the process of deciphering the captchas—that is, they saw stars progressively appearing on the screen to indicate that the captcha was being solved. To ensure that they based their judgements on the stimulus parameters that we were manipulating, participants were not shown the captchas except for one example in the tutorial. The tutorial captcha consisted of six characters and were deciphered in 14 s.

Participants estimated others’ effort costs of deciphering a captcha on a Likert-type scale (1–7), where 1 means effortless and 7 means effortful.

#### Procedure

Participants were informed that they would be participating in a task in which they would have to watch recordings of people solving captchas. They were informed that they would complete eight trials in total and that they would estimate others’ effort costs of deciphering a captcha on a Likert-type scale (1–7), where 1 means effortless and 7 means effortful. The eight trials were preceded by a tutorial video in which stars progressively appeared to indicate that the partner was solving a captcha and upon completion the captcha key was revealed. At the end of the experiment, participants had to answer three comprehension check questions. Then participants were debriefed and paid.

#### Design

In a within-subject design experiment, we manipulated the process of deciphering the captcha by two factors: path length and speed/time. We manipulated Length by modifying the number of steps (characters) it takes to solve the captcha. There were four levels of captcha path length: 3, 6, 10, and 12 steps. In addition, we manipulated the Speed/Time at which these steps were taken. Captchas of the same length were completed twice as fast in the Fast condition than in the Slow condition.

To estimate the partner’s effort costs in deciphering the captchas, participants used a Likert-type scale (1–7), where 1 means effortless and 7 means effortful.

#### Data preparation and analysis

We prepared and analysed the data in rStudio (RStudio Team, 2016) using R 4.0.0 (R Core Team, 2020), the *tidyverse* (v1.3.0; Wickham et al., 2019), the *rjags* (*v4-10*; Martyn Plummer, 2019), the *HH* (*v3.1-47*; Heiberger & Holland, 2022), and the *runjags* (*v2.0.4-6*; Denwood, 2016) packages.

For the Bayesian data analysis, we used a noncommittal broad prior on the parameters so that the prior had minimal influence on the posterior. We used Markov chain Monte Carlo (MCMC) techniques to generate representative credible values from the joint posterior distribution on the parameters (Kruschke, 2015). Three chains were initialised, well burned in (for 1,000 steps), and a total of 30,000 steps were saved. The chains were checked for convergence and autocorrelation and run long enough to produce an effective sample size (ESS) of at least 10,000 for all of the reported results. This yielded a stable and accurate representation of the posterior distribution on the parameters.

### Results

**Figure 3.**
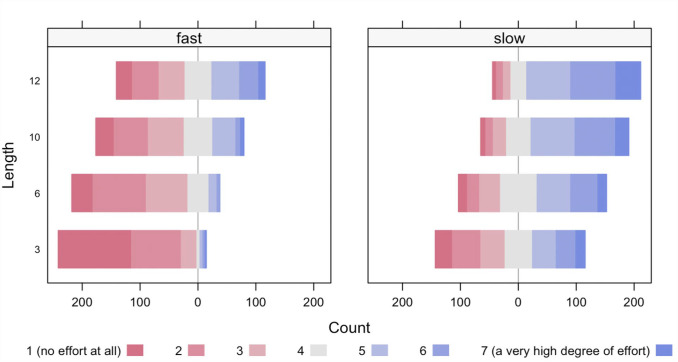
We depicted how participants rated others’ effort costs of deciphering a captcha on a Likert-type scale (1–7) (where 1 means “no effort at all” and 7 means “a very high degree of effort”) on a multidimensional frequency plot.

We examined how participants rated others’ effort costs in deciphering a captcha as a function of Length and Speed/Time with a two-way ordinal regression (see Figure 3 and Table 1). The results revealed a significant main effect of Length, χ^2^(3) = 353.297, *p* < .001, that is, participants rated more steps (captchas consisting of more characters) as more effortful, a significant main effect of Speed/Time, χ^2^(1) = 517.704, *p* < .001, that is, participants rated actions as more effortful in the Slow condition than in the Fast condition, and also a significant interaction term, χ^2^(3) = 18.62, *p* < .001, that is, the effect of Length was greater in the Fast condition than in the Slow condition. Moreover, pairwise comparisons showed that the perceived effort costs were significantly different between each level of the factor Length.

**Table 1.** Median and IQR for the ordinal ratings at each level of the factors.

Length	Speed	
	Fast	Slow
3	2 (1)	4 (2)
6	3 (2)	4 (1.5)
10	3 (2)	5 (2)
12	4 (2)	5 (2)

We examined the data with Bayesian methods as well. We used a generalised linear model, in which the predicted value is described as categorically distributed around a linear combination of nominal predictors (Speed/Time, Length, random effect of participant) mapped to a probability value via a thresholded cumulative normal function. The results revealed a main effect of Speed/Time, a main effect of Length, and an interaction effect on participants’ ratings. Accordingly, the credible values of the difference between Slow and Fast had a mode of −1.72 and a 95% HDI (the 95% highest density interval contains the most credible 95% of the values) that extended from −1.83 to −1.6; hence, zero falls outside of this range and accordingly deemed not credible (because we modelled the distribution of ordinal values with an underlying metric variable, we report the credible values on the underlying metric scale and not on the response scale of ordinal ratings or on the probability scale). This means that participants rated slow action as more effortful than fast action. The credible values of the difference of 3 steps and 12 steps had a mode of −1.83 and a 95% HDI that extended from −1.96 to −1.66; zero was accordingly deemed not credible. Pairwise comparisons showed that perceived effort costs were different between each level of the factor Length, that is, more steps were rated as more effortful. The credible values of the interaction effect had a mode of −0.693 and a 95% HDI that extended from −0.981 to −0.382; zero deemed not credible, that is, the effect of Length was greater in the Fast condition than in the Slow condition.

## Experiment 2

In Experiment 1, we found that participants rated others’ effort costs in deciphering a captcha as a function of Length and Speed/Time. Specifically, they rated more steps (captchas consisting of more characters) as more effortful, and they rated slow action as more effortful than fast action. Moreover, the effect of Length was greater in the Fast condition than in the Slow condition.

It is important to note that speed and time were confounded in Experiment 1. We manipulated speed by manipulating the time at which the steps were taken to solve the captcha. This means that the main effect of speed was simultaneously a main effect of time, because for each level of the factor Length, the slower action always lasted longer than the faster action. In other words, it is impossible to compare fast actions and slow actions, and in doing so to keep path length constant, without simultaneously comparing longer and shorter durations. However, if one compares *across* the levels of the factor Length, the situation is different. To see this, consider the following. The first-level captcha consisted of three characters and was deciphered in 8 s in the Slow condition. The third-level captcha consisted of 10 characters and was deciphered in 8 s in the Fast condition. Critically, the former was rated as more effortful than the latter even though they were of the same time and the latter consisted of more steps. Thus, our results suggest that speed can have an independent effect on people’s judgement on others’ effort costs—regardless of the effect of time or path length.

However, the conjecture that speed has an independent effect on people’s judgement on others’ effort costs has not yet been directly tested or confirmed—it is merely supported by an exploratory comparison of two conditions. To further investigate the separate effects of speed and time on participants’ judgement on others’ effort costs, we ran a second experiment. In doing so, we manipulated Time by modifying the number of seconds it takes to solve the captcha (8.7 s, 13.51 s, 17.48 s) and we manipulated Speed/Length: each level of Time was completed with two path lengths, that is, twice as many steps had to be taken in the Fast condition as in the Slow condition. We predicted a main effect of Time and a main effect of Speed/Length.

### Method

#### Participants

Our target sample was 200 participants as in Experiment 1. We collected data from 208 participants. Of these, five individuals were excluded from analyses because they did not complete the task or failed two of three comprehension check questions, leaving a sample of 203 (i.e., 203 participants: 80 female, *M*_age_ = 28.03 years, *SD*_age_ = 10.11 years) participants in the final data set. All participants were recruited through the Prolific recruitment platform (www.prolific.co) and were naive to the purpose of the study. All participants gave their informed consent at the start of the experiment, could withdraw from the experiment at any time, and received a fee of 80 pence for their participation. The experiment was conducted in accordance with the Declaration of Helsinki and was approved by the (EPKEB) United Ethical Review Board for Research in Psychology.

#### Apparatus and stimuli

The apparatus and stimuli were identical to that of Experiment 1 except for the following.

There were six videos in which stars progressively appeared to indicate that an agent was solving a captcha. The first-level captchas were deciphered in 8.7 s and consisted of three characters in the Slow condition and six characters in the Fast condition. The second level captchas were deciphered in 13.51 s and consisted of six characters in the Slow condition and 12 characters in the Fast condition. The third-level captchas were deciphered in 17.48 s and consisted of 12 characters in the Slow condition and 24 characters in the Fast condition.

#### Procedure

The procedure was identical to that of Experiment 1.

#### Design

In a within-subject design experiment, we manipulated the process of deciphering the captcha by two factors: Time and Speed/Length. We manipulated Time by modifying the number of seconds it takes to solve the captcha. There were three levels of Time: 8.7, 13.51, and 17.48 s. In addition, we manipulated Speed/Length: each level of Time was completed with two path lengths, that is, twice as many steps had to be taken in the Fast condition than in the Slow condition. The dependent measure was identical to that of Experiment 1.

#### Data preparation and analysis

The data preparation and analysis were identical to that of Experiment 1.

### Results

**Figure 4.**
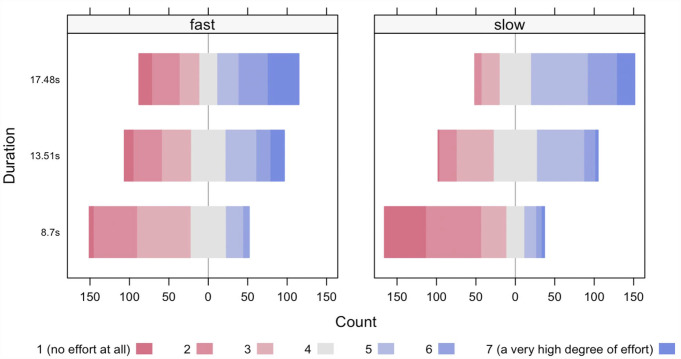
Depiction of how participants rated others’ effort costs of deciphering a captcha on a Likert-type scale (1–7) (where 1 means “no effort at all” and 7 means “a very high degree of effort”) on a multidimensional frequency plot.

We examined how participants rated others’ effort costs in deciphering a captcha as a function of Time and Speed/Length with a two-way ordinal regression (see Figure 4 and Table 2). The results revealed a significant main effect of Time, χ^2^(2) = 232.581, *p* < .001, that is, longer time was rated as more effortful, no main effect of Speed/Length, χ^2^(1) = 1.784, *p* = .181, and a significant interaction term, χ^2^(2) = 34.802, *p* < .001. Specifically, we found three different effects of Speed/Path length depending on the level of Time, that is, participants rated fast action as more effortful than slow action when the duration was 8.7 s, participants rated fast action and slow action similarly when the duration was 13.51 s, and participants rated slow action as more effortful than fast action when the duration was 17.48 s. Moreover, pairwise comparisons showed that perceived effort costs were significantly different between each level of the factor Time.

**Table 2.** Median and IQR for the ordinal ratings at each level of the factors.

Duration	Speed	
	Fast	Slow
8.7	3 (2)	3 (2)
13.51	4 (2)	4 (2)
17.48	4 (2.25)	5 (1)

We examined the data with Bayesian methods as well. We used a generalised linear model, in which the predicted value is described as categorical distributed around a linear combination of nominal predictors (Speed/Length, Time, random effect of participant) mapped to a probability value via a thresholded cumulative normal function. The results revealed no main effect of Speed/Length, a main effect of Time, and an interaction effect on participants’ ratings. Accordingly, the credible values of the difference of Slow and Fast had a mode of 0.0863 and a 95% HDI that extended from −0.066 to 0.22; zero was deemed credible (because we modelled the distribution of ordinal values with an underlying metric variable, we report the credible values on the underlying metric scale and not on the response scale of ordinal ratings or on the probability scale). The credible values of the difference of 8.7 s and 17.48 s had a mode of −0.624 and a 95% HDI that extended from −0.842 to −0.474; zero was deemed not credible. Pairwise comparisons showed that perceived effort costs were different between each level of Time, that is, longer time was rated as more effortful. The credible values of the interaction effect had a mode of 1.28 and a 95% HDI that extended from 0.92 to 1.66; zero was deemed not credible.

## General discussion

Effort perception is a crucial capacity underpinning characteristically human forms of sociality, allowing us to learn about others’ mental states and about the value of opportunities afforded by our environment, and supporting our ability to cooperate efficiently and fairly. Across two experiments, we provide new insight into how people estimate the cognitive effort costs that observed agents are investing in specific ongoing activities. In Experiment 1, we found that participants rated others’ effort costs in deciphering a captcha as a function of Length and Speed/Time. Specifically, they rated more steps (captchas consisting of more characters) as more effortful, and for each level of the factor Length they rated slow action as more effortful than fast action. Moreover, the effect of Length was greater in the Fast condition than in the Slow condition. Importantly, in Experiment 1, we could not cleanly separate the effect of speed and time because, within each level of the factor Length, the slower action always lasted longer than the faster action—in other words, the main effect of Speed was also a main effect of Time. However, when looking across levels of the factor Length, we were able to compare faster and slower actions with the same duration (i.e., with different path lengths). This analysis revealed that slower actions were perceived as more effortful than faster actions even when the time was constant. Building on this finding in Experiment 2, we manipulated Time and Speed/Length independently. We found a main effect of Time, that is, longer time was rated as more effortful, no main effect of Speed/Length and an interaction effect. Specifically, we found three different effects of Speed/Length depending on the level of Time, that is, at the level of the shortest time, fast action was rated more effortful than slow action; at the middle level time, fast action was rated similarly to slow action; and at the level of the longest time, fast action was rated as less effortful than slow action. Critically, in Experiment 2, we could not separate the effect of Speed and Length because, for each level of the factor Time, the faster action always consisted of more steps than the slower action. This means that Length did not have a main effect on effort perception either. This is in contrast to the results of Experiment 1, where we found a main effect of Length. Across the two experiments, only Time had a consistent effect on effort perception, that is, participants rated longer time as more effortful. Taken together, our results suggest that within the context of our task—observing an agent deciphering a captcha—people rely on the time of others’ action to estimate others’ cognitive effort costs.

Why did participants interpret longer time as an indication of higher level of cognitive effort? One possibility is that the way people process movement cues with respect to estimating others’ effort costs depends on the task and other contextual cues. For example, in our task participants saw stars progressively and continuously appearing on the screen that might have been interpreted as a sign of engaged attention and therefore the continuous investment of cognitive effort. However, our stimuli could be modified so that time would not necessarily correspond to attentional engagement. For example, the stars could begin appearing on the screen and then stop, followed by a long pause after which stars continue appearing and the captcha is completed—signalling attentional disengagement and the cessation of cognitive effort in the middle of the action. In this case, participants may not interpret the longer time as a sign of a higher level of effort. Alternatively, it may be that people have a general expectation that greater magnitude in time covaries with greater outlays of energy—regardless of contextual cues. If so, we should expect to find this effect of time across a wide range of tasks. Thus, future research should investigate whether people differentiate between different kinds of time: time of engaged and disengaged attention, or more generally whether people’s perception of others’ cognitive effort costs through movement cues depends on contextual factors.

The current findings contribute to previous research in at least three ways. First, they provide a crucial test of assumptions about effort perception made by a large body of work using movement cues as a basis for effort perception. By probing the mechanisms by which people estimate the effort costs invested by observed agents, they provide an important addition to recent research on the computational principles governing the attribution of goals, preferences, and other mental states as well as abilities to observed agents, based on the costs and benefits of their actions (i.e., the “naive utility calculus,” Jara-Ettinger, 2016).

Second, we tested whether the principles gained from experiments implementing physical effort costs can be extended to situations in which adults have the task to perceive cognitive effort through perceptible properties of action. To our knowledge, the experiments reported here are the first to directly test how adults perceive others’ cognitive effort costs. The difference in perceiving cognitive or physical effort is important because cognitive and physical effort differ characteristically in their appearance to an observer. For example, when an agent does not exert a high degree of physical force, it is appropriate to judge them to be exerting a low level of physical effort or no physical effort at all—however, they may be still exerting a high level of cognitive effort, such as inhibiting impulses, maintaining a task set or engaging in mental planning. Accordingly, our findings suggest that participants appraised slowness as indicative of high cognitive effort regardless of time, although this was not a consistent effect. Further research is needed to investigate the differences in how we perceive cognitive and physical effort.

Third, our findings also complement existing research on how people compare the relative difficulty of different kinds of tasks. For example, Gray et al. (2006) found that participants chose between perceptual-motor strategies and cognitive strategies as a function of time to minimise time on task. Building on these results, Potts et al. (2018) and Rosenbaum and Bui (2019) invited participants to choose between a counting task and a bucket-carrying task. They found that relative task duration predicted participants’ choices. These results suggest that people use time as a common currency to compare the relative difficulty of different kinds of tasks. Extending these results, we provide evidence that time is a key source of information that people use to draw inferences in attributing effort investment to others.

The present study raises key questions for future research. For example: What is the functional form of the relationship between time and the perception of others’ effort costs? A linear model predicts a constant effect of duration on effort perception regardless of the absolute value of duration. But there are other possibilities. For instance, a hyperbolic model predicts that changes in short duration have a stronger impact than changes in long duration. In contrast, a parabolic model predicts the opposite: Changes in long duration have a stronger impact than changes in short duration. In sum, these three functions differ in their assumptions on how increasing duration impacts effort perception. Identifying the relevant functional form is important insofar as it would enable us to design more precise stimuli for various research programmes that build on our ability to perceive others’ effort. Future research should address this question by developing theories that make precise predictions about the form of this function, and empirically distinguishing among them.

Moreover, in our study, we focused on the systematic differences in how people rate others’ effort costs in deciphering a captcha. However, our study did not speak to the accuracy of these ratings. An interesting next step would be to test this by correlating participants’ ratings of others’ effort costs with those other agents’ own internal assessment of their effort investment.

In our study, participants were told that they would view a series of brief videos that had been recorded of a person solving captchas. It must be acknowledged that we did not ask participants about whether they in fact believed that the videos really did depict other humans trying to decipher captchas. On the basis of previous results, we have reason to believe that participants do in fact believe that this is the case. Specifically, using the same stimuli, Székely and Michael (2018) found that participants calibrated their own effort investment in response to the apparent effort investment of their partner when they were informed (as in the present study) that the partner was a human (Experiment 1) but not when they were informed that the partner investing effort was an algorithm. Interestingly, it has also been shown, again using the same stimuli, that if people believe that the agent is a humanoid robot, they respond as if the partner were a human (Székely et al., 2019). Future research should systematically investigate factors influencing people’s willingness to attribute effort to other agents.

Finally, our findings provide support for the hypothesis that people perceive others’ effort costs by tracking perceptible properties of movement. However, there are at least two other hypotheses about the sources of information and mechanisms operating on them that may enable us to perceive others’ effort. First, building on results suggesting that during observation of an action, a corresponding representation in the observer’s cortical motor system is activated (Frith & Singer, 2008; Rizzolatti & Craighero, 2004; Barchiesi & Cattaneo, 2015), it may be fruitful to explore the possibility that we perceive others’ effort through our own motor system. Second, one may speculate that we estimate effort costs by tracking perceptible properties of others’ autonomic nervous systems such as breathing patterns and cues of muscle tension, because cues to the level of activity of the autonomic nervous system convey information about the current level of effort investment (Rejeski & Lowe, 1980; De Morree & Marcora, 2010). Critically, these mechanisms of effort perception are mutually compatible and may or may not interact in a number of different ways. Further research is needed to distinguish among these hypotheses and to clarify how we integrate these various sources of information.

## References

[bibr1-17470218231183963] Apps M. A. Rushworth M. F. Chang S. W. (2016). The anterior cingulate gyrus and social cognition: Tracking the motivation of others. Neuron, 90(4), 692–707. 27196973 10.1016/j.neuron.2016.04.018 PMC4885021

[bibr2-17470218231183963] Barchiesi G. Cattaneo L. (2015). Motor resonance meets motor performance. Neuropsychologia, 69, 93–104. 25619846 10.1016/j.neuropsychologia.2015.01.030

[bibr3-17470218231183963] Chennells M. Michael J. (2018). Effort and performance in a cooperative activity are boosted by perception of a partner’s effort. Scientific Reports, 8, 15692. 30356160 10.1038/s41598-018-34096-1 PMC6200738

[bibr4-17470218231183963] Csibra G. (2008). Goal attribution to inanimate agents by 6.5-month-old infants. Cognition, 107(2), 705–717. 17869235 10.1016/j.cognition.2007.08.001

[bibr5-17470218231183963] Csibra G. Bíró S. Koós O. Gergely G. (2003). One-year-old infants use teleological representations of actions productively. Cognitive Science, 27(1), 111–133.

[bibr6-17470218231183963] Csibra G. Gergely G. Bíró S. Koos O. Brockbank M. (1999). Goal attribution without agency cues: The perception of “pure reason” in infancy. Cognition, 72(3), 237–267. 10519924 10.1016/s0010-0277(99)00039-6

[bibr7-17470218231183963] De Morree H. M. Marcora S. M. (2010). The face of effort: Frowning muscle activity reflects effort during a physical task. Biological Psychology, 85(3), 377–382. 20832447 10.1016/j.biopsycho.2010.08.009

[bibr8-17470218231183963] Denwood M. J. (2016). Runjags: An R package providing interface utilities, model templates, parallel computing methods and additional distributions for MCMC models in JAGS. Journal of Statistical Software, 71(9), 1–25. 10.18637/jss.v071.i09

[bibr9-17470218231183963] Frith C. D. Singer T. (2008). The role of social cognition in decision making. Philosophical Transactions of the Royal Society B: Biological Sciences, 363(1511), 3875–3886. 10.1098/rstb.2008.0156 PMC2581783 18829429

[bibr10-17470218231183963] Gergely G. Csibra G. (2003). Teleological reasoning in infancy: The naïve theory of rational action. Trends in Cognitive Sciences, 7(7), 287–292. 12860186 10.1016/s1364-6613(03)00128-1

[bibr11-17470218231183963] Gergely G. Nádasdy Z. Csibra G. Bíró S. (1995). Taking the intentional stance at 12 months of age. Cognition, 56(2), 165–193. 7554793 10.1016/0010-0277(95)00661-h

[bibr12-17470218231183963] Gray W. D. Sims C. R. Fu W. T. Schoelles M. J. (2006). The soft constraints hypothesis: A rational analysis approach to resource allocation for interactive behavior. Psychological Review, 113(3), 461. 16802878 10.1037/0033-295X.113.3.461

[bibr13-17470218231183963] Heiberger R. M. Heiberger M. R. M. (2022). Package ‘HH’.

[bibr14-17470218231183963] Ibbotson P. Hauert C. Walker R. (2019). Effort perception is made more accurate with more effort and when cooperating with slackers. Scientific Reports, 9(1), 1–8. 31767878 10.1038/s41598-019-53646-9 PMC6877554

[bibr15-17470218231183963] Jara-Ettinger J. Gweon H. Schulz L. E. Tenenbaum J. B. (2016). The naïve utility calculus: Computational principles underlying commonsense psychology. Trends in Cognitive Sciences, 20(8), 589–604. 27388875 10.1016/j.tics.2016.05.011

[bibr16-17470218231183963] Jara-Ettinger J. Gweon H. Tenenbaum J. B. Schulz L. E. (2015). Children’s understanding of the costs and rewards underlying rational action. Cognition, 140, 14–23. 25867996 10.1016/j.cognition.2015.03.006

[bibr17-17470218231183963] Kamewari K. Kato M. Kanda T. Ishiguro H. Hiraki K. (2005). Six-and-a-half-month-old children positively attribute goals to human action and to humanoid-robot motion. Cognitive Development, 20(2), 303–320.

[bibr18-17470218231183963] Kruschke J. (2015). Doing Bayesian data analysis: A tutorial with R, JAGS, and Stan (2nd ed.). Elsevier Science.

[bibr19-17470218231183963] Leonard J. A. Lee Y. Schulz L. E. (2017). Infants make more attempts to achieve a goal when they see adults persist. Science, 357(6357), 1290–1294. 28935806 10.1126/science.aan2317

[bibr20-17470218231183963] Liang Y. Wolf T. Török G. Székely M. Michael J. (2019). Comparing effort perception in individual and joint action contexts. https://osf.io/b7stn/

[bibr21-17470218231183963] Liu S. Ullman T. D. Tenenbaum J. B. Spelke E. S. (2017). Ten-month-old infants infer the value of goals from the costs of actions. Science, 358(6366), 1038–1041. 29170232 10.1126/science.aag2132

[bibr22-17470218231183963] Martyn Plummer. (2019). rjags: Bayesian graphical models using MCMC [R package version 4-10]. https://CRAN.R-project.org/package=rjags

[bibr23-17470218231183963] Michael J. Liang Y. Wolf T. Török G. Székely M. (2019). Comparing effort perception in individual and joint action contexts. https://osf.io/b7stn/

[bibr24-17470218231183963] Peirce J. W. (2007). PsychoPy—Psychophysics software in Python. Journal of Neuroscience Methods, 162(1–2), 8–13. 17254636 10.1016/j.jneumeth.2006.11.017 PMC2018741

[bibr25-17470218231183963] Potts C. A. Pastel S. Rosenbaum D. A. (2018). How are cognitive and physical difficulty compared? Attention, Perception, & Psychophysics, 80(2), 500–511. 10.3758/s13414-017-1434-2 29116613

[bibr26-17470218231183963] R Core Team. (2020). R: A language and environment for statistical computing. R Foundation for Statistical Computing. https://www.R-project.org/

[bibr27-17470218231183963] Rejeski W. J. Lowe C. A. (1980). Nonverbal expression of effort as causally relevant information. Personality and Social Psychology Bulletin, 6(3), 436–440.

[bibr28-17470218231183963] Rizzolatti G. Craighero L. (2004). The mirror-neuron system. Annual Review of Neuroscience, 27, 169–192. 10.1146/annurev.neuro.27.070203.144230 15217330

[bibr29-17470218231183963] Rosenbaum D. A. Bui B. V. (2019). Does task sustainability provide a unified measure of subjective task difficulty? Psychonomic Bulletin & Review, 26(6), 1980–1987. 31240614 10.3758/s13423-019-01631-8

[bibr30-17470218231183963] RStudio Team. (2016). RStudio: Integrated development for R. RStudio. http://www.rstudio.com/

[bibr31-17470218231183963] Southgate V. Johnson M. H. Csibra G. (2008). Infants attribute goals even to biomechanically impossible actions. Cognition, 107(3), 1059–1069. 18078920 10.1016/j.cognition.2007.10.002

[bibr32-17470218231183963] Székely M. Michael J. (2018). Investing in commitment: Persistence in a joint action is enhanced by the perception of a partner’s effort. Cognition, 174, 37–42. 29407604 10.1016/j.cognition.2018.01.012

[bibr33-17470218231183963] Székely M. Michael J. (2023). In it together: Evidence of a preference for the fair distribution of effort in joint action. Evolution and Human Behavior. Advance online publication. 10.1016/j.evolhumbehav.2023.04.002

[bibr34-17470218231183963] Székely M. Powell H. Vannucci F. Rea F. Sciutti A. Michael J. (2019). The perception of a robot partner’s effort elicits a sense of commitment to human-robot interaction. Interaction Studies, 20(2), 234–255.

[bibr35-17470218231183963] Verschoor S. Biro S. (2012). Primacy of information about means selection over outcome selection in goal attribution by infants. Cognitive Science, 36(4), 714–725. 22141746 10.1111/j.1551-6709.2011.01215.x

[bibr36-17470218231183963] Wickham H. Averick M. Bryan J. Chang W. D’Agostino McGowan L. François R. Grolemund G. Hayes A. Henry L. Hester J. Kuhn M. Pedersen T. L. Miller E. Bache S. M. Müller K. Ooms J. Robinson D. Seidel D. P. Spinu V. . . .Yutani H. (2019). Welcome to the tidyverse. Journal of Open Source Software, 4(43), 1686. 10.21105/joss.01686

[bibr37-17470218231183963] Woodward A. L. (1998). Infants selectively encode the goal object of an actor’s reach. Cognition, 69(1), 1–34. 9871370 10.1016/s0010-0277(98)00058-4

